# Determination of Species Composition of Mosquitoes in Lahore, Pakistan

**DOI:** 10.18502/jad.v14i1.2717

**Published:** 2020-03-31

**Authors:** Farkhanda Manzoor, Robeela Shabbir, Madiha Sana, Sumbal Nazir, Muhammad Aslam Khan

**Affiliations:** 1 Lahore College for Women University, Lahore College for Women University, Jail Road, Lahore, Pakistan; 2 Department of Zoology, Lahore College for Women University, Lahore, Pakistan; 3 University of Health Sciences Lahore, Lahore, Pakistan

**Keywords:** Mosquito fauna, Lahore, Shannon Index, Simpson Index, Climatic factors

## Abstract

**Background:**

Present study was conducted to determine species composition of mosquitoes (larvae, pupae and adults) collected from ten different towns of Lahore from September 2014 to August 2015.

**Methods:**

Mosquito larvae, pupae and adults (male and female) were collected by using dippers and aspirators from September 2014 to August 2015 in different sites of Lahore comprising of ten towns i.e. Iqbal, Aziz Bhatti, Data Ganj Baksh, Gulberg, Nishtar, Ravi, Samanabad, Shalimar, Wagah, and Lahore Cantonment. Mosquito larvae and adults were identified by standard entomological keys. Diversity, richness and rarity of mosquito fauna were analyzed by the Shannon, Simpson and Margalef indices respectively.

**Results:**

In this study, a total of 8656 mosquitoes belonging to four genera namely *Anopheles*, *Culex*, *Aedes* and *Mansonia* were identified. Among fifteen species collected, *Cx. quinquefasciatus* was the most abundant species in the city having 25.8% relative abundance. However *An. culicifacies s.l.* (*sensu lato*) was reported as the least abundant species with 0.22% relative abundance. The highest diversity of mosquitoes was shown in the month of August (H= 2.25) while the lowest diversity was recorded June (H= 1.43). Extensive sewage water supported the maximum abundance of *Cx. quinquifasciatus* in urban areas of this city.

**Conclusions:**

This study has significantly elaborated the monthly varying species composition of mosquito fauna of this city. Hence this research will help us to find out the control strategies of mosquito borne diseases in this region.

## Introduction

Mosquito serves as a vector of various deadly diseases including Malaria, Dengue fever, Zika fever and Chikungunya. The vector causes deaths of more than one million people annually (1). Worldwide there are about 3541 species of mosquitoes reported belonging to 42 different genera (2). However there are 134 species of mosquitoes found in Pakistan belonging to Anophelinae and Culicinae subfamilies (3). Mosquitoes play an important role in food chains of various ecosystems as well (4).

Pakistan is one of those subtropical countries that have been under threat of vector borne diseases. Like other Asian countries, Pakistan is also withstanding substantial climatic changes that are sufficient to harbor the outbreaks of mosquito borne diseases (5). Lahore, an urbanized city of Pakistan, was reported to have 11,283 cases of Dengue fever in the epidemics of 2012 (6). Recent Dengue fever outbreaks in Khyber Pakhtunkhwa resulted in 2179 positive cases (7).

The shift in the climate of South Asian countries tends to escalate the incidence of mosquito borne diseases (8). Moreover, the abiotic factors like temperature, humidity and rainfall are positively supporting the population of mosquitoes with increased sustainability of their breeding sites and long survivorship (9, 10).

Likewise, certain studies have been conducted in various regions of Pakistan reporting the mosquito fauna that have related the relative abundance of different species with the gradual shift in climatic trend (11–15). The relationship between the biodiversity of mosquitoes in Lahore with its climatic conditions was studied in 2013. The reported species were *Aedes aegypti*, *Culex quinquefasciatus*, *Anopheles stephensi* and *Anopheles subpictus* s.l.. The population dynamics of these mosquito species was strongly correlated with seasonal temperature variations of the region (15). The rapid urbanization of this city with extensive road construction projects and elimination of open fields is a reason to provide the flourishing breeding sites for disease transmitting mosquito species. Hence, it is so crucial to monitor the abundance of various mosquito species for maintaining vector control programs in this region.

The objective of current study was to describe the abundance, diversity and rarity of different species of mosquitoes in different habitat types. Morever this study would also find a link between environmental variables and the relative abundance of different species of mosquitoes collected.

## Materials and Methods

**Fig. 1.**
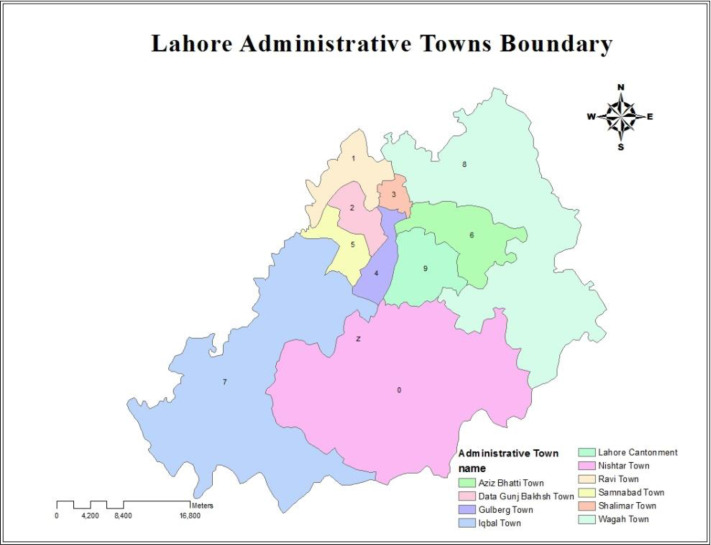
Location of study sites in towns of urban city Lahore, Pakistan, September 2014 to August 2015

Lahore having a semi-arid climate and typical monsoon season was selected as the area of study. The geological coordinates of Lahore are 31° 32′ North and 74° 20′ East respectively and is about 223 meter high from sea level. The selected towns of this city were Iqbal Town, Aziz Bhatti Town, Data Ganj Baksh Town, Gulberg Town, Nishtar Town, Ravi Town, Samanabad Town, Shalimar Town, Wagah Town and Lahore Cantonment as shown in the Lahore Administrative Towns Boundary (Fig. 1).

### Sampling of larvae, pupae and adult mosquitoes

Mosquito samples were collected from fixed spots of ten towns four times in a month from September 2014 to August 2015. Mosquito larvae and pupae were collected by using strainers and dippers (16) from fresh as well as polluted water bodies of all towns (17). Simultaneously, indoor and outdoor collection was done for adult mosquitoes by using sweeper and aspirators (14). Open drains, freshwater reservoirs, discarded containers, flower vases, sewage water, tyres and tree-holes were keenly observed during sampling of larvae, pupae and adult mosquitoes.

The collected mosquitoes were transferred to plastic jars covered with net for further experimentation.

### Preservation and identification of the mosquitoes

The adult mosquitoes were sucked through aspirator from different plastic jars and were then kept in airtight conical jars containing chloroform dipped cotton swab that helped in killing the adult mosquitoes in two to four minutes. These killed mosquitoes were shifted in test tubes with a cork containing small amount of silica gel for preservation of the mosquitoes. The collected larvae and pupae were identified under the dissecting microscope.

The mosquito larvae, pupae and killed adults were later identified using standard taxonomic keys given in “The fauna of British India including Ceylon and Burma, Diptera Family Culicidae, Tribe Anopheline, Vol. IV and Tribe Megarhinini and Culicini, Vol. V” (18, 19).

### Data analysis

Relative abundance was determined by using following formula.

$$\text{Relative}\;\text{abundance}=\frac{n}{\text{Total}\;\text{number}\;\text{of}\;\text{species}}\times 100$$

Where ‘n’ is the number of mosquito, and it is divided by the total number of mosquitoes collected and then it is multiplied by 100 (20). The mosquito species were termed as dominant species with more than 10% relative abundance and subdominant species with 3 to 10% relative abundance while mosquitoes species having lesser than 3% relative abundance were tagged satellite species (21).

The distribution status of collected mosquito species in different collection sites was calculated by the formula as given (21),

Distribution(C)=n×100/N

Where n is number of sites where species was found, and N is the total number of sites. The distribution status of different species was categorized as constant species (80–100%), frequent species (60–80%), moderate species (40–60%) and infrequent species (20–40%).

Mosquito species richness was calculated by using Margalef’s index of richness (Dmg)

Dmg=(S−1)/ln N.

Where, S is total number of species and N is total number of individuals.

Shannon-Weaver Index was also applied to find out the diversity of mosquitoes with respect to monthly variation (22).

$$\text{H}=-\sum {\text{p}}_{\text{i}}{\text{lnp}}_{\text{i}}$$

where H= Shannon diversity index and

$${\text{p}}_{\text{i}}=\text{ni}/\text{N}$$

Where ni= importance value index of the species and N= importance value index of all species.

Simpson index (D) was also applied to find out the rarity of mosquito species (23) collected. It was calculated by using formula,

D=Σn(n−1)\N(N−1)

Whereas, n= no. of mosquitoes of individual species, and N= No. of total mosquitoes species collected.

### Climate analysis

Annual reports of climate data from September 2014 to August 2015 were retrieved from Punjab Metrological Department for the analysis of temperature, humidity and rainfall. The collected data were further processed by plotting different graphs on MS Excel. Moreover, Poisson regression analysis was also performed on SPSS 16.0 to develop a correlation between the frequency of mosquitoes collected and changing weather variables.

## Results

Mosquito species were recovered from the different types of particular habitat such as open drains, fresh water, discarded containers, flower vases, sewage water, tyres and tree holes (Table 1). *Anopheles* species and *Mansonia* species was specifically found to be Fresh water. *Culex* species were collected from their breeding sites of fresh water, discarded containers and sewage water. *Aedes* species were collected from different sites except sewage water, all of its breeding sites were predominately fresh water in nature.

**Table 1.** Different breeding habitats of mosquitoes in ten different towns of Lahore, Pakistan, September 2014 to August 2015

**Mosquito**	**Open drains**	**Fresh water**	**Discarded Containers**	**Flower vases**	**Sewage water**	**Tyres**	**Tree Holes**
***An. annularis***	**−**	**+**	**−**	**−**	**−**	**−**	**−**
***An. pulcherrimus***	**−**	**+**	**−**	**−**	**−**	**−**	**−**
***An. subpictus* s.l.**	**−**	**+**	**−**	**−**	**−**	**−**	**−**
***An. nigerrimus***	**−**	**+**	**−**	**−**	**−**	**−**	**−**
***An. stephensi***	**−**	**+**	**−**	**−**	**−**	**−**	**−**
***An. culicifacies* s.l.**	**−**	**+**	**−**	**−**	**−**	**−**	**−**
***Cx. quinquefasciatus***	**+**	**−**	**+**	**−**	**+**	**−**	**−**
***Cx. tritaeniorhynchus***	**+**	**−**	**+**	**−**	**+**	**−**	**−**
***Cx. vagans***	**+**	**−**	**+**	**−**	**+**	**−**	**−**
***Cx. vishnui***	**−**	**−**	**+**	**−**	**+**	**−**	**−**
***Cx. theileri***	**+**	**−**	**+**	**−**	**+**	**−**	**−**
***Cx. sitiens***	**+**	**−**	**+**	**−**	**+**	**−**	**−**
***Ae. aegypti***	**+**	**+**	**+**	**+**	**−**	**+**	**+**
***Ae. albopictus***	**+**	**+**	**+**	**+**	**−**	**+**	**+**
***Ma. uniformis***	**−**	**+**	**−**	**−**	**−**	**−**	**−**

Relative abundance of fifteen different species of mosquitoes throughout Lahore with their abundance status and distribution class was shown in Table 2. *Culex quinquefasciatus* was the most abundant species with 25.80% relative abundance. In contrast *An. culicifacies* was appeared to be the least in number with 0.22% relative abundance. *Culex sitiens* and *Cx. tritaeniorhynchus* were reported as dominant species during collection. Moreover, *An. pulcherrimus*, *Cx. vagans, Cx. theileri*, *Cx. vishnui*, *Ae. albopictus* and *Ae. aegypti* were found sub dominant species. However, *An. annularis*, *An. nigerrimus*, *An. subpictus* s.l., *An. stephensi*, and *An. culicifacies* were recovered as satellite species having least abundance and sporadic distribution. *Mansonia uniformis* was found to be infrequent species in its distribution.

**Table 2.** Relative abundance, status and distribution class of mosquitoes collected from all the ten towns of Lahore, Pakistan, September 2014 to August 2015

**Species**	**Abundance**	**Relative Abundance**	**Relative Abundance Status**	**Distribution class**
***An. annularis***	217	2.51	Satellite	Sporadic
***An. pulcherrimus***	308	3.56	Satellite	Sporadic
***An. subpictus* s.l.**	166	1.92	Satellite	Sporadic
***An. nigerrimus***	150	1.73	Satellite	Sporadic
***An. stephensi***	51	0.59	Satellite	Sporadic
***An. culicifacies* s.l.**	19	0.22	Satellite	Sporadic
***Cx. sitiens***	1029	11.89	Dominant	Sporadic
***Cx. quinquefasciatus***	2233	25.80	Most dominant	Moderate
***Cx. tritaeniorhynchus***	868	10.03	Dominant	Moderate
***Cx. vagans***	795	9.18	Subdominant	Moderate
***Cx. vishnui***	141	1.63	Satellite	Moderate
***Cx. theileri***	810	9.36	Subdominant	Moderate
***Ae. albopictus***	962	11.11	Dominant	Frequent
***Ae. aegypti***	730	8.43	Subdominant	Frequent
***Ma. uniformis***	177	2.04	Satellite	Infrequent

In this study, total of 8656 mosquitoes were collected. Taxonomic identification revealed fifteen species of mosquitoes such as *An. annularis*, *An. pulcherrimus*, *An. stephensi*, *An. nigerrimus*, *An. subpictus* s.l., *An. culicifacies*, *Cx. vagans*, *Cx. quinquefasciatus*, *Cx. sitiens*, *Cx. tritaeniorhynchus*, *Cx. vishnui*, *Cx. theileri*, *Ae. aegypti*, *Ae. albopictus* and *Ma. uniformis.* An overview of month wise abundance of all mosquito species collected in one year of study was shown in Table 3.

**Table 3.** Richness and abundance of mosquito population in Lahore, Pakistan, September 2014 to August 2015

**Month**	***An. annularis***	***An. pulcherrimus***	***An. subpictus* s.l.**	***An. nigerrimus***	***An. stephensi***	***An. culicifacies* s.l.**	***Cx. sitiens***	***Cx. quinquefasciatus***	***Cx. tritae-niorhynchus***	***Cx. vagans***	***Cx. vishnui***	***Cx. theileri***	***Ae. albopictus***	***Ae. aegypti***	***Ma. uniformis***	**Total**
**Sep-2014**	90	130	75	68	23	10	209	593	197	168	33	179	255	194	52	2276
**Oct-2014**	37	44	24	21	3	0	64	147	43	33	0	0	4	0	0	420
**Nov-2014**	0	0	0	0	0	0	0	0	0	0	0	0	0	0	0	0
**Dec-2014**	0	0	0	0	0	0	0	0	0	0	0	0	0	0	0	0
**Jan-2015**	0	0	0	0	0	0	0	0	0	0	0	0	0	0	0	0
**Feb-2015**	0	0	0	0	0	0	0	0	0	0	0	0	0	0	0	0
**Mar-2015**	0	0	0	0	0	0	0	0	0	0	0	0	0	0	0	0
**Apr-2015**	0	0	0	0	0	0	167	314	141	152	28	162	138	108	34	1244
**May-2015**	0	0	0	0	0	0	160	341	123	131	23	165	146	122	25	1236
**Jun-2015**	0	0	0	0	0	0	40	100	85	26	0	35	0	0	0	286
**Jul-2015**	0	0	0	0	0	0	154	239	104	107	18	110	180	130	30	1072
**Aug-2015**	90	134	67	61	25	9	235	499	175	178	39	159	239	176	36	2122
**Total**	217	308	166	150	51	19	1029	2233	868	795	141	810	962	730	177	8656

**Fig. 2.**
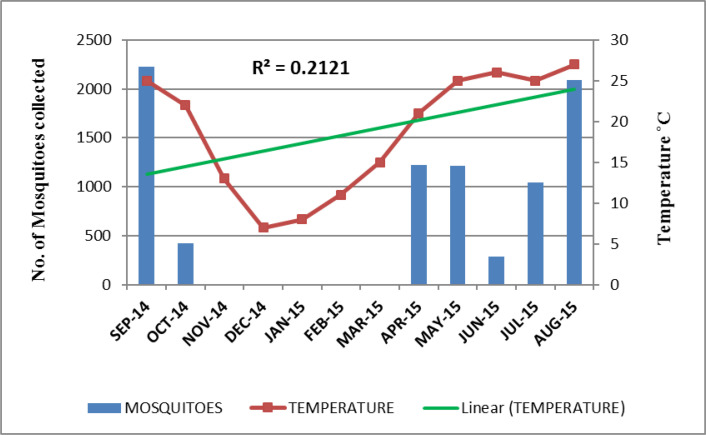
Relationship between temperature and the total number of mosquitoes collected monthly in Lahore, Pakistan, September 2014 to August 2015

**Fig. 3.**
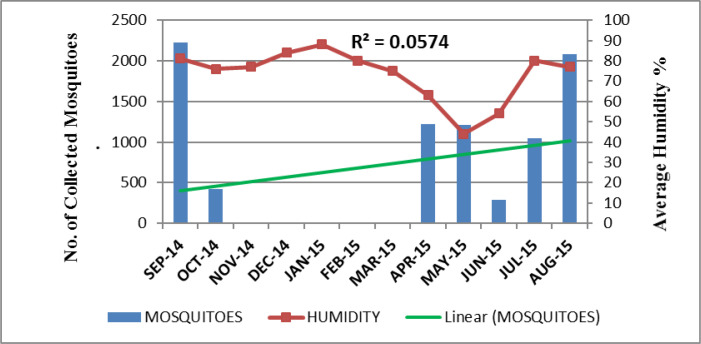
Relationship between the average humidity and the total number of mosquitoes collected monthly in Lahore, Pakistan, September 2014 to August 2015

**Fig. 4.**
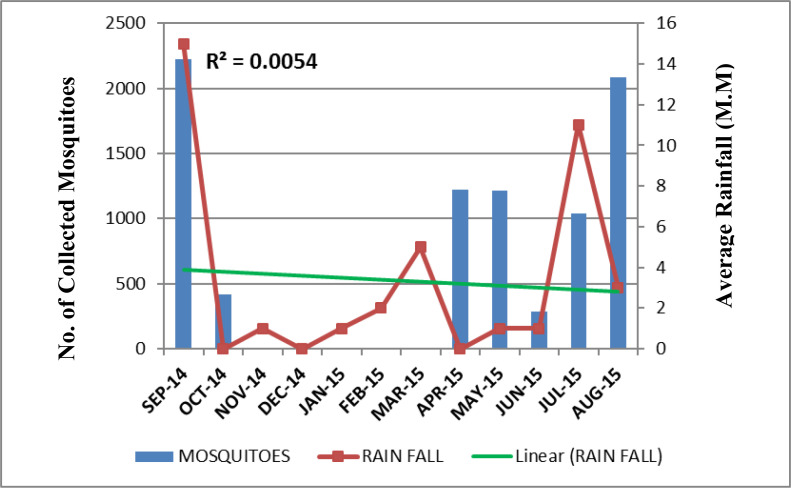
Relationship between rainfall and the total number of mosquitoes collected monthly in Lahore, Pakistan, September 2014 to August 2015

The highest mosquito sample size was shown in the month of September (Dmg= 4.17) while the lowest sample size was shown in June (Dmg= 1.63). On the basis of Shannon index, the highest diversity was shown in August (H= 2.24) while the lowest diversity was shown in June (H= 1.43). Due to the highest diversity of mosquitoes in August, less number of rare species of mosquitoes (D= 0.11) was present. The lowest diversity in June showed the highest number of rare species (D= 0.25) in this month (Table 4). The relationship of seasonal conditions recorded in every month with the abundance of mosquitoes collected was shown in (Figs. 2–4).

**Table 4.** Monthly analysis of species composition of mosquitoes collected from different towns of Lahore, Pakistan, September 2014 to August 2015

**Months**	**Abundance**	**Margalef’s Index (Dmg)**	**Shannon index (H)**	**Simpson Index (D)**
**September**	2276	4.17	2.15	0.12
**October**	420	3.43	1.82	0.18
**November**	0	0	0	0
**December**	0	0	0	0
**January**	0	0	0	0
**February**	0	0	0	0
**March**	0	0	0	0
**April**	1244	2.58	1.93	0.15
**May**	1236	2.59	1.91	0.16
**June**	286	1.63	1.43	0.25
**July**	1072	2.64	1.92	0.14
**August**	2122	2.40	2.24	0.11

Table 5 enlisted the results of Poisson regression analysis showing the correlation between various climatic variables and expected number of mosquitoes to be collected.

**Table 5.** Parameter estimates of Poisson regression analysis showing correlation between weather variables and expected mosquito frequency from different towns of Lahore, Pakistan, September 2014 to August 2015

	**Estimate**	**Standard Errors**	**Exp (B)**	**P- value**
**Intercept**	2.038	.0068	7.677	.000
**Temperature**	−0.012	.0020	0.989	.000
**Humidity**	0.010	.0021	1	.000
**Rainfall**	0.021	.0009	1.021	.000

## Discussion

Fifteen species of mosquitoes belonging to four genera *Anopheles*, *Aedes*, *Culex* and *Mansonia* were recovered from the urban city Lahore during the period of September 2014 to August 2015.

Firstly there were twenty nine species of mosquitoes ever reported in Lahore, Pakistan (24). In 1971, the mosquito fauna of this city was highly enriched with the identification of 31 different species belonging to seven genera (7). However, current study exhibited reduction in the number of collected mosquito species. This decline in reported mosquito species of this city during last forty years is due to drastic change in environmental conditions, expanding sewage system and due to diminishing freshwater habitats. Mosquito collection performed in this study by dippers and aspirator mainly focused on indoor and outdoor breeding sites. However by employing other methods such as, ovitrap, CDC light traps, BG traps, human and animal baits, the species composition of the region can modulate accordingly.

*Culex quinquefasciatus* was the most abundant species found in all the towns with the highest diversity. Same species was also reported as the most dominant species of Pakistan in a study analyzing the mosquito diversity based on barcoding (5). This species breeds in polluted water therefore it is found in sewage canal which is an air conditioned breeding place for this mosquito.

Mukhtar et al. (2003) investigated the breeding of mosquitoes in waste water irrigation where he found three genera *Aedes*, *Culex* and *Anopheles.* The present study discovered nearly all the species such as *An. pulcherrimus*, *An. culicifacies* s.l., *An. subpictus* s.l., *An. stephensi*, *Cx. quinquefasciatus* and *Cx. tritaeniorhynchus* which were previously collected from waste-water (25).

Ali and Rashid (26) studied polluted water of Palosai stream near Peshawar. They identified *Cx. quinquefasciatus* and *An. stephensi* while *Aedes* was missing. This shows that *Aedes* species are the inhabitants of temporary habitats only. By using different traps and trapping techniques twelve different mosquito species were collected in Lahore in 2013 (15). In our study *An. subpictus* s.l., *An. culicifacies* s.l., and *Ma. uniformis* were additionally reported.

*Anopheles culicifacies* s.l. and *An. stephensi* are fresh water breeders. They have almost disappeared from main city and are recovered from suburb areas getting more susceptible to diseases like malaria. *Anopheles annularis* and *An. nigerrimus* are usually found in clean and still water. The species were recovered from all the ten towns of Lahore. Both of the species are considered as vector of malaria in India, Nepal and Srilanka (27). The risk of transmission of malaria by these species is quite low, because in Pakistan there is no evidence of their vectorial capacity. However further studies can be conducted to investigate these species as suspected vectors of malaria in this region. The disappearance of fresh water in this city has lowered the relative abundance of fresh water dwelling species like *An. pulcherrimus* and *An. subpictus* s.l. thus enlisting them as sporadic in distribution*.*

*Aedes* species were largely taken from temporary habitats over the entire city in suitable breeding sites of fresh water like water tanks, broken pots, pans and old tyres that explain the vulnerability of Dengue epidemics in Lahore.

In our study, the recorded optimum temperature, 25 °C, optimum humidity, 80% and 15mm rainfall in the months having maximum number of species built the positive correlation between the climatic conditions and abundance of mosquitoes collected. The Poisson regression analysis in table 5 proposed that for every unit increase in temperature the expected mosquito frequency increased by e^−0.012^ = 0.988. Likewise, for every unit increase in rainfall and humidity, collected mosquito number would be increased by e^0.21^ = 1.233 and e^0.01^ = 1.01 respectively. The climatic analysis of this study showed the most suitable environmental conditions like temperature, humidity and rainfall in the month of August and September that is referred as the typical monsoon period in Pakistan. Along with suitable climatic variables for survival, this season also provides extensive breeding sites for different species of mosquitoes. The results of this study also exhibited fresh water sources, discarded containers and open drains as more productive breeding sites for dwelling mosquito species and all of these sites are more functioning in the months of monsoon.

The highest mosquito diversity (H= 2.24) was reported in August due to the availability of high rainfall and suitable humidity. This ultimately enhances the dissemination of vector borne diseases. Due to the highest diversity of mosquitoes in August, the least number of rare species were recorded. Likewise, the highest species richness was found in September (Dmg= 4.17). In these months, high temperature in water also boosts larval development making the survival of mosquitoes more feasible (28).

## Conclusions

From the findings of this study and previous studies it can be concluded that a worldwide climatic shift along extensive urbanization is influencing the distribution as well as the diversity of mosquito fauna of this cosmopolitan city. Hence, there is always a potential risk of the outbreak of the mosquito borne diseases. The results of this research provide significant help to plan effective control strategies against mosquito borne diseases before time. Moreover, it is further suggested to plan similar studies by employing other methods of mosquito collection to assess current species composition in Lahore, Pakistan.
